# Interdisciplinary Innovations Utilizing Pet Robots to Meet Research, Education, and Care Needs

**DOI:** 10.1093/geroni/igab046.3502

**Published:** 2021-12-17

**Authors:** Meredith Troutman-Jordan, Boyd Davis

**Affiliations:** 1 University of North Carolina Charlotte, Charlotte, North Carolina, United States; 2 UNC Charlotte, Charleston SC, South Carolina, United States

## Abstract

Studies of the impact of robotic companion pets are proliferating, authored by several disciplines, each with different concerns. Roboticists focus on technology design and artificial emotional intelligence as opposed to general preferences for soft, furry, interactive animals. Others worry that as people interact with potentially deceptive technology, they may think the pet is alive. While aware of these serious concerns, gerontologists have focused on how lonely older persons without cognitive impairment respond to social ‘helper’ robots. More recent studies emphasize the possible impact of animatronic pets on persons with dementia (PWD). Therapeutic benefits of these pets are just being established. Our current pilot study is timely in that it now involves semi-structured interviews with formal/ informal caregivers of PWD who have been given a robot pet. We are eliciting perceptions, opinions, and observations of the PWD’s response to robotic pets. We recruited 8 gerontology students as much-needed assistants for a research-driven topics course to afford them field exposure to PWD, caregivers, and direct research experience. Because students seldom have experience either with robotic pets or PWD, they read selected articles and received training/practice in semi-structured interviewing techniques. Students next conducted interviews with caregivers of PWD who have interacted with the pets. All interviews are audio-recorded, transcribed and deposited in the Carolinas Conversations Collection. Content and thematic analysis of transcriptions, student activity logs and bi-weekly reflective discussions will inform next steps in intervention research, testing therapeutic outcomes such as agitation reduction by pet robots for PWD.

